# Development of Simple Sequence Repeats (SSR) Markers in *Setaria italica* (Poaceae) and Cross-Amplification in Related Species

**DOI:** 10.3390/ijms12117835

**Published:** 2011-11-11

**Authors:** Heng-Sheng Lin, Chih-Yun Chiang, Song-Bin Chang, Chang-Sheng Kuoh

**Affiliations:** Department of Life Sciences, Institute of Biodiversity, National Cheng Kung University, Tainan 70101, Taiwan; E-Mails: t58601109520@gmail.com (H.-S.L); chyuchiang@gmail.com (C.-Y.C); kuohpopo@gmail.com (C.-S.K.)

**Keywords:** model system, *Setaria italica*, SSRs, Taiwan, transferability

## Abstract

Foxtail millet is one of the world’s oldest cultivated crops. It has been adopted as a model organism for providing a deeper understanding of plant biology. In this study, 45 simple sequence repeats (SSR) markers of *Setaria italica* were developed. These markers showing polymorphism were screened in 223 samples from 12 foxtail millet populations around Taiwan. The most common dinucleotide and trinucleotide repeat motifs are AC/TG (84.21%) and CAT (46.15%). The average number of alleles (*N*_a_), the average heterozygosities observed (*H*_o_) and expected (*H*_e_) are 3.73, 0.714, 0.587, respectively. In addition, 24 SSR markers had shown transferability to six related Poaceae species. These new markers provide tools for examining genetic relatedness among foxtail millet populations and other related species. It is suitable for germplasm management and protection in Poaceae.

## 1. Introduction

Foxtail millet (*Setaria italica* (L.) P. Beauv) is an old cereal consumed by people in Euraisa, Americas, Africa and Australia. It has also been an important crop for the indigenous tribes of Taiwan for thousands of years. It is able to endure droughts and grows quickly; therefore, the indigenous people cultivate foxtail millet instead of rice. Though several researches have used a few millet strains from Taiwan, little is known about the genetic diversity in hundreds of local landraces scattered throughout Taiwan [1,2].

With a relatively small genome (515 Mb), the diploid foxtail millet has been adopted as model organism for providing deep understanding of the plant biology [3]. The importance of this species is rising since the project of its genome draft has been conducted by the Joint Genome Institute (JGI) of the US Department of Energy [4]. Compared to other familiar model systems, such as Arabidopsis (*Arabidopsis thaliana*), rice, or maize, large amounts of the genetic information were provided as an important resource for the research community. However, insufficient genetic resources of *Setaria italica* distributed around the world have been studied.

Several molecular markers in foxtail millets have been utilized including RFLP [1,3], RAPD [5], AFLP [6], and simple sequence repeats (SSR) [7,8]. These researches mainly focused on the species origin and the genetic map construction; no studies were conducted to assess the genetic diversity of the varying landraces in Taiwan.

Microsatellite, which is also called simple sequence repeats (SSR), are tandem repeat sequences of 1–6 base pairs of DNA. It has been proven to be useful in genetic diversity studies because of its high polymorphism, high variation, with abundant information and convenience; thus it is widely employed in many species [9–14]. Broadening the genetic base is regarded as a major task in species where inbreeding works have resulted in the decline of genetic diversity [15]. Several SSR markers have been developed by Jia *et al*. [7–8], as no one can ensure if novel SSR markers provide benefits or not. Reports have demonstrated that tetranucleotides are typically easier to be genotyped than di- and tri-nucleotide repeat SSRs [16]. To distinctly quantify the genetic variation among varying foxtail millets landraces in Taiwan, more SSR loci need to be developed.

In the present study, we report the identification of different types of SSR loci within representative landraces of foxtail millet by examining their polymorphism and cross-amplification in a further six related Poaceae species.

## 2. Results and Discussion

### 2.1. Microsatellite Loci Isolation

A total number of 570 genomic sequences from the RAPD-enriched library and 158 genomic sequences downloaded from the GenBank were screened for SSRs. In these sequences, 134 SSR among the 570 sequences and 16 SSR from the 158 GenBank sequences were found. A total of 150 SSR primer pairs were designed successfully by PRIMER3. Forty-five out of 150 SSR markers showed polymorphism among seven millets strains (NCKU.S.I.P1001, NCKU.S.I.P2001, NCKU.S.I.P3001, NCKU.S.I.P4001, NCKU.S.I.P5001, NCKU.S.I.P6001, NCKU.S.I.P7001) (Table 1).

### 2.2. Characterization of Microsatellite Loci

Among these 45 SSRs, 19 contained dinucleotide repeats, and 26 contained trinucleotide. The most common dinucleotide repeats motif was AC/TG (84.21%). CAT was the most common trinucleotide repeat motif in foxtail millet (46.15%). However, according to research of EST-SSR markers of foxtail millet, the most common repeat motifs were TC/AG [7] and CAG/TCT [8]. The most common repeats in wheat are CA or TG, also GA [17] or GT repeats [18]. In other monocot crops such as barley, wheat, maize, sorghum and rice, the most common trinucleotide repeats were CCG/GGC or AAC/TTG [19]. The differences may be caused by different genomes being tested or our use of different SSR isolation strategies with varying affinities.

### 2.3. Genetic Characterization of Microsatellite Loci

Forty-five SSRs were PCR amplified in 223 samples from 12 collection sites to assess the diversity of foxtail millet in Taiwan. Characterizations of these loci are summarized in Table 2. The average number of alleles (*N*_a_) ranged from 1 to 8, with an average of 3.73. The average observed heterozygosities (*H*_o_) ranged from 0 to 0.886 with an average of 0.714. The expected heterozygosities (*H*_e_) ranged from 0 to 0.813, with an average of 0.587. No linkage disequilibrium was observed from pairwise comparisons of loci. 32 SSR loci significantly deviated from Hardy-Weinberg equilibrium (*H*_W_) (Table 2 and Table 1S), which were assumed to be a result of long time isolation of the foxtail millet population in Taiwan. GenBank (BLASTX) searches indicated that three SSR loci among the 45 SSR markers, including SITM 15, SITM34 and SITM59 have putative function at E values less than 10^−05^ (Table 1).

### 2.3. Cross-Amplification of SSR Loci in Related Poaceae Species

Cross-species amplification with the 45 SSR primers obtained from *Setaria italica* were applied to six other related species (*N* = 18), including *Hygroryza aristata* (Retz.) Nees (Asian watergrass), *Setaria plicata* (Lamk.) *T cooke* (Small palm grass), *Microstegium vimineum* (Trin.) A camus (Flexible sasa grass), *Oplimenus compositus* (L.) P. Beauv (Armgrass), *Cynodon dactylon* (L.) Pers (Bermuda Grass), and *Setaria verticillata* (L.) P. Beauv (Hooked Bristlegrass). Finally, 24 primers could yield PCR products in the other Poaceae species, indicating transferability of the markers (53%) (Table 3).

## 3. Experimental Section

### 3.1. Samples Collection

A total of 223 samples of *Setaria italica* were collected from 12 sites in Taiwan. Six Poaceae species each with 3 samples were collected for cross-species amplification. All samples of the tested materials were listed in Table 4. Genomic DNA was isolated from leaf tissue of each individual using a DNeasy Plant Mini Kit (Qiagen, Hilden, Germany).

### 3.2. Development and Screening of SSR Markers

The strategies in this study are based on PCR isolation of microsatellite arrays (PIMA), which began with an enriched pool of small DNA fragments amplified using RAPD primers [20]. PCR amplification was performed in 20 μL volume containing 20 ng of genomic DNA, 0.2 mM of dNTPs, 2 mM MgCl_2_, 0.2 U Go-Taq polymerase (Promega, Madison, Wisconsin, USA), and 5 pmol of one RAPD primers. Five-hundred RAPD primers were used to construct randomly amplified fragments library (MDBIO, Piscataway, New Jersey, USA). Reactions were run on an MyCyclerTM Thermal Cycler (BIO-RAD, Benicia, California, USA) using the following conditions: 3 min of denaturation at 94 °C, followed by 45 cycles at 94 °C for 1 min, annealing temperature specific to each primer for 1 min, extension at 72 °C for 2 min, and a final extension at 72 °C for 5 min. Amplification products were analyzed in the electropHoresis (2% agarose gel using 100-bp ladder molecular size standard) (Geneaid, Taipei, Taiwan) to evaluate the allele size through ethidium bromide staining. The amplified DNA fragments with the size of 200–2000 bp were extracted using the Gel Extraction kit (Geneaid, Taipei, Taiwan). DNA fragments were ligated into a p-GEM-T Easy Vector following the manufacturer’s instruction and the plasmids were transformed into *Escherichia coli* cells (Promega, Madison, Wisconsin, USA). Each clone was screened using repeat-specific primers including (AC)_5_, (AG)_5_, (AT)_5_, (CG)_5_, (CT)_5_ and (GT)_5_ and 2 vector primers including forward M13 (5′-dGTTTTCCCAGTCACGAC-3′) and reverse M13 (5′-dGTTTTCCCAGTCACGAC-3′) primers. The conditions of colony-PCR are 3 min at 94 °C, followed by 45 cycles at 94 °C for 1 min, annealing at 53 °C for 1 min, 2 min at 72 °C, and 5 min at 72 °C. In positive clones, a DNA fragment which contains a SSR appears as a band on the gel. Plasmid DNA of positive clones was purified using the Plasmid Miniprep Kit (BioKit, Miaoli, Taiwan). Ten μL of plasmid DNA with a concentration of 100 ng/μL was used in each sequencing reaction. DNA sequencing in both directions of the insert DNA was conducted using an Applied Biosystems 3730 DNA Analyzer with BigDyeR Terminator v3.1 Cycle Sequencing Kit (Applied Biosystems, Foster, California, USA). There were 134 SSRs among 570 positive clones examined.

To obtain additional useful SSR markers, 158 genomic DNA sequences of foxtail millets were downloaded from GenBank [21]. The criteria of no less than 16 repeat units for mono-, nine for di-, five for tri- to hexa-nucleotide repeats in perfect SSR and no less than 12 bp for imperfect SSR were adopted. Finally, a total of 150 SSR primer pairs were designed using PRIMER3 software for detecting the diversity of foxtail millet in Taiwan [22].

### 3.3. Characterization of Developed SSR Primers

To evaluate the usefulness of SSRs, PCR reactions were performed on 20 μL volumes containing 10 ng of genomic DNA, 0.2 mM dNTPs, 2 mM MgCl_2_, 0.2 U Go-Taq polymerase and 0.12 μm of each primer. PCR conditions were as follows: 3 min at 94 °C, followed by 35 cycles at 94 °C for 1 min, annealing temperature specific to each primer for 1 min, 1 min at 72 °C, and 5 min at 72 °C.

The average number of allele (Na), the average observed (Ho), and expected heterozygosities (He) were calculated using the software CERVUS 3.0 [23]. Test of deviation of Hardy-Weinberg equilibrium (*H*_W_) and linkage disequilibrium (LD) were performed using the GenePop program [24]. The sequences were searched against the GenBank nucleotide collection database using TBLASTX for functional annotation with a thresHold of E-value < 1.00 E^−05^.

### 3.4. Cross-Amplification of Developed SSR Markers

Furthermore, cross-species amplification of the SSR primers obtained from *Setaria italica* were applied to six other related species (*N* = 18). SSR markers were PCR amplified on 20 μL volumes containing 10 ng of genomic DNA, 0.2 mM dNTPs, 2 mM MgCl_2_, 0.2 U Go-Taq polymerase and 0.12 μm of each primer. The conditions are carried out as following: 3 min at 94 °C, 35 cycles at 94 °C for 1 min, annealing temperature specific to each primer for 1 min, 1 min at 72 °C, and 5 min at 72 °C.

## 4. Conclusions

In summary, these 45 novel SSR markers of foxtail millet showed polymorphism and transferability to the related Poaceae species in Taiwan. They can be used as molecular markers for application of population genetics, breeding and further landraces identification.

## Supplementary Material



## Figures and Tables

**Table 1:** Polymorphic simple sequence repeats (SSR) primers for *Setaria italica*.

Locus	Repeat Sequence	Primer Sequence (5′-3′)	Ta (°C)	Size	Accession No.	OS	PH
SITM02	(TG)_12_	F TAGTCGCTGGAAAGTTTCGG R TAGTCGCTGGAAAGTTTCGG	51.8	208	JN565177	–	No hit
SITM04	(TG)_13_	F CGTGTCCTTGTACTCAGCCA R CAATGGTCTCAGGTGTGGTG	53.8	240	JN565179	–	No hit
SITM05	(GT)_10_	F AGCTTACCCCTCACATTTAT R ATGAGAAGGTGCCAAAATGC	47.7	204	JN565180	–	No hit
SITM06	(CA)_10_	F GCTCTCTCCATCCCACATTC R TTCTCCTTCCCTTCCTTTCC	53.8	146	JN565181	–	No hit
SITM07	(AC)_7_.(CA)_8_	F GCCCAAAAACTCATTCTCCA R ATAACCCTCACCACTACAAG	49.7	55	JN565182	–	No hit
SITM09	(AC)_15_	F CCCCTATGTTCCTTGGACCT R GGAAAGCCAGTGTGAGTGCA	53.8	207	JN565184	–	No hit
SITM10	(TCA)_5_	F GGCTGGAGTGAGTCTTCGTC R GCTGAGGAAAATGGTGAGGA	55.9	178	JN565185	–	No hit
SITM11	(ATC)_6_	F CTCGCCCATCTCTTCTTCAG R CAAGCACAGGGAAGAGGAGT	53.8	113	JN565186	–	No hit
SITM14	(CAT)_15_	F TCTGAGGAGGAGGATGTGCT R CATCTGAAGCAAACCTGAAT	53.8	196	JN565189	–	No hit
SITM15	(ATC)_8_	F TGGAACCGAAGCTGCCTACC R AAGTCCAAGAAGTCGCCAGA	55.9	223	JN565190	–	Sorghum bicolor hypothetical protein
SITM17	(AG)_10_	F GCATACGGCTACTGGACATA R ATCTTCTTTTGTTAGCGAGC	51.8	109	JN565192	–	No hit
SITM18	(CAT)_8_	F GCTCGCTAACAAAAGAAGAT R AGGTTGAAATGAAGAAGAGG	47.7	72	JN565193	–	No hit
SITM19	(TCA)_8_	F CTTCCGCCATCAACCATTCG R GACGAAGATGATGACGACGA	53.8	63	JN565194	–	No hit
SITM20	(TGA)_6_	F TGATGATGCCAATGAACCAG R GCTATTTCCTACGCCCTTCC	49.7	246	JN565195	–	No hit
SITM22	(ATG)_7_	F TCCAAGTAGTGAAAGTGATA R TTCCTCCTCGTCCTCTTCAT	45.6	188	JN565197	–	No hit
SITM23	(ATG)_5_	F ATGAAGAGGACGAGGAGGAA R CGTTCCAGTAATATGTGCCC	51.8	110	JN565198	–	No hit
SITM24	(ATG)_7_	F AGGTCTGCTTGGGATGAAAT R AACATTACCCCCTGAAGAAC	47.7	110	JN565199	–	No hit
SITM25	(ATC)_6_	F CTCGCCCATCTCTTCTTCAG R CAAGCACAGGGAAGAGGAGT	53.8	113	JN565200	–	No hit
SITM26	(TGA)_15_	F TGAAGCAAACCTGAATCGTG R TCTGAGGAGGAGGATGTGCT	49.7	186	JN565201	–	No hit
SITM27	(CAT)_20_	F TTTACAGCCAAGGAAGACGT R GCTCCTCGATGGTATGCTCT	49.7	221	JN565202	–	No hit
SITM28	(TGA)_5_	F TAAGATGAGCGTTGGGGAGA R ACGAACCGCACCAAATCTAC	51.8	101	JN565203	–	No hit
SITM30	(ATG)_7_.(ATG)_6_	F TGTCGGAGATGATGAGGTGA R GACGAACCGCATCAAATCTAA	51.8	220	JN565205	–	No hit
SITM32	(GAT)_6_	F CAGGATGACCAGGGAGATGC R ACAGCTTTCCGCCTCAACCT	55.9	157	JN565207	–	No hit
SITM33	(ATC)_9_	F TTTGGACGACAGACGATTCA R AAGTCCAAGAAGTCGCCAGA	49.7	160	JN565208	–	No hit
SITM34	(CAT)_5_	F AAGGGGTGGATGAGGTAGGT R TCGAATTGAAGAAGAGCCTG	53.8	147	JN565209	–	Sorghum bicolor hypothetical protein
SITM37	(GAT)_7_	F CATCGTTGTAAGAAGTGGAA R CTTTTTGGCTGCTGGGTTT	47.7	166	JN565212	–	No hit
SITM38	(TCA)_9_	F ACGGAAGAGGCAGTCACAAT R ATTGGTGATGGATTCGTCAT	51.8	206	JN565213	–	No hit
SITM40	(ATC)_9_	F GTTGCTGCTGATGCTTGGT R AATGCGAATCTCTTGGTGCT	51.1	219	JN565215	–	No hit
SITM41	(ATC)_5_	F GGTTTCCTTCCCCTTGTGTT R CGGTCCCTATTGTTGATGAT	51.8	87	JN565216	–	No hit
SITM42	(ATG)_8_	F TGTTCATGCGGATTTTCTTG R GGGACTCGGCAAAATAATCA	47.7	169	JN565217	–	No hit
SITM44	(TTA)_5_	F TCGGTTAATGCCTTTTGCTC R TTATGGACGGAAATGGTGTG	49.7	70	JN565219	–	No hit
SITM46	(TGA)_6_	F TGCCGAAAGGATCAAAAAGA R TCACCACTGCCATCATCACT	47.7	215	JN565221	–	No hit
SITM49	(TG)_10_.(GT)_18_	F AGGTATCGTGCGTGTGTCTG R AATGATGAATAATGGTGCTG	53.8	82	JN565224	–	No hit
SITM51	(AC)_13_	F CAATGGTCTCAGGTGTGGTG R TACCATTCATCAAAAGTGCC	53.8	164	JN565226	–	No hit
SITM53	(GT)_9_.(GT)_10_	F GTCACTTGTTGTTGTTGCGA R GAACACGGAGAAGCGAAAAG	49.7	158	JN565228	–	No hit
SITM55	(AC)_14_	F GTCGTAGCTTTCGGTCCAAC R CTGGGAATAGAAGAACATGC	53.8	196	JN565230	–	No hit
SITM57	(AC)_13_	F GGGTAGTGGTCTGGTGGTCA R GTATCACTTCAGGCGGCATT	55.9	196	JN565232	–	No hit
SITM59	(TG)_22_	F AGGAAGGGGAAACACTGACC R GCGTTGTTGTTCATCGTGTT	53.8	158	JN565234	–	Glycine max isolate RG10 lipoxygenase 2 (Lx2)
SITM62	(AC)_15_.(AC)_6_	F CGAACCGCTCACAAACACTA R TAGTTGGAGAAGTTGAGTGC	51.8	149	JN565237	–	No hit
SITM65	(GT)_13_	F GCCACCCCTTGATTGTTATG R GCTCAACATCTGGCATTTCA	51.8	228	JN565240	–	No hit
SITM68	(GT)_26_	F GGCATTGGACGAGTTACGGC R GTCATAGCTCACGGCACAAC	55.9	108	JN565243	–	No hit
SITM73	(CT)_21_	F CCTGAACTGGTTGGAGTTGG R ATCAGGACCAAGGGCAAAAT	53.8	243	JN565248	–	No hit
SITM84	(CT)_3_.(AT)_3_.(GA)_4_. (GA)_7_.(GGC)_3_	F TCGGTCCTTCACCTTCTTTG R CGCCATCACCTTCTCCTCGC	51.8	110	JN565259	EF117799	No hit
SITM86	(AT)_3_.(CG)_3_.(CG)_3_. (CGC)_7_.(CG)_3_	F CTTGCTTAGATCTGGACTAA R GCGAGGCTGGAGATAGTCAG	47.7	202	JN565261	EF117797	No hit
SITM91	(GGC)_4_	F GTTCGCAGCAGCACTCATTA R TTGCATGTGCAGGTATAGGC	51.8	161	JN565266	–	No hit

F: forward primer; R: reverse primer; Ta: annealing temperature; Size: expected size of PCR products (bp); OS: original sequences retrieved from Genbank; PH: putative homology.

**Table 2:** Results of diversity estimation in 223 samples of *Setaria italica* in Taiwan.

Locus	*N*_a_	*H*_o_	*H*_e_	*H*_W_	Locus	*N*_a_	*H*_o_	*H*_e_	*H*_W_
SITM02	5	0.701	0.75	**	SITM33	3	0.76	0.625	ND
SITM04	7	0.883	0.647	***	SITM34	7	0.781	0.653	ND
SITM05	6	0.886	0.64	***	SITM37	1	0	0	ND
SITM06	3	0.786	0.533	***	SITM38	2	0.828	0.371	ND
SITM07	4	0.726	0.607	***	SITM40	2	0.753	0.653	ND
SITM09	5	0.757	0.629	***	SITM41	1	0	0	ND
SITM10	3	0.744	0.736	***	SITM42	4	0.773	0.52	***
SITM11	3	0.719	0.75	***	SITM44	7	0.774	0.61	***
SITM14	6	0.783	0.639	***	SITM46	3	0.723	0.57	***
SITM15	4	0.788	0.62	***	SITM49	8	0.801	0.687	***
SITM17	3	0.792	0.75	***	SITM51	7	0.875	0.653	***
SITM18	2	0.704	0.813	**	SITM53	4	0.673	0.625	ND
SITM19	3	0.783	0.764	ND	SITM55	6	0.707	0.653	***
SITM20	2	0.873	0.653	***	SITM57	6	0.683	0.575	***
SITM22	2	0.76	0.667	**	SITM59	3	0.74	0.75	***
SITM23	2	0.726	0.625	ND	SITM62	3	0.743	0.652	***
SITM24	3	0.782	0.778	***	SITM65	3	0.747	0.588	***
SITM25	3	0.702	0.694	ND	SITM68	2	0.757	0.487	***
SITM26	1	0	0	***	SITM73	4	0.731	0.563	***
SITM27	2	0.838	0.569	ND	SITM84	4	0.817	0.736	***
SITM28	7	0.75	0.468	**	SITM86	2	0.69	0.478	***
SITM30	2	0.794	0.468	**	SITM91	4	0.722	0.625	ND
SITM32	4	0.783	0.542	ND					

** *p* < 0.05;

*** *p* < 0.01; ND: non-significant deviation.

**Table 3:** Cross-amplification in six related Poaceae species.

Locus	A	B	C	E	F	G	Locus	A	B	C	E	F	G
SITM02	−	−	−	−	−	−	SITM33	−	−	−	−	−	−
SITM04	+	+	+	+	+	+	SITM34	−	−	−	−	−	−
SITM05	+	+	+	+	+	+	SITM37	−	−	−	−	−	−
SITM06	+	+	+	+	+	+	SITM38	+	+	+	+	+	+
SITM07	+	+	+	+	+	+	SITM40	−	−	−	−	−	−
SITM09	+	+	+	+	+	+	SITM41	+	+	+	+	+	+
SITM10	+	+	+	+	+	+	SITM42	−	−	−	−	−	−
SITM11	+	+	+	+	+	+	SITM44	−	−	−	−	−	−
SITM14	−	−	−	−	−	−	SITM46	−	−	−	−	−	−
SITM15	−	−	−	−	−	−	SITM49	+	+	+	+	+	+
SITM17	+	+	+	+	+	+	SITM51	−	−	−	−	−	−
SITM18	+	+	+	+	+	+	SITM53	−	−	−	−	−	−
SITM19	+	+	+	+	+	+	SITM55	−	−	−	−	−	−
SITM20	+	+	+	+	+	+	SITM57	−	−	−	−	−	−
SITM22	+	+	+	+	+	+	SITM59	−	−	−	−	−	−
SITM23	+	+	+	+	+	+	SITM62	+	+	+	+	+	+
SITM24	+	+	+	+	+	+	SITM65	+	+	+	+	+	+
SITM25	+	+	+	+	+	+	SITM68	−	−	−	−	−	−
SITM26	+	+	+	+	+	+	SITM73	−	−	−	−	−	−
SITM27	+	+	+	+	+	+	SITM84	−	−	−	−	−	−
SITM28	−	−	−	−	−	−	SITM86	+	+	+	+	+	+
SITM30	−	−	−	−	−	−	SITM91	+	+	+	+	+	+
SITM32	−	−	−	−	−	−							

+: successful amplification with expected allele size; −: absence of amplification; A: *Hygroryza aristata* (Retz.) Nees; B: *Setaria plicata* (Lamk.) T cooke; C: *Microstegium vimineum* (Trin.) A camus; E: *Oplimenus compositus* (L.) P. Beauv; F: *Cynodon dactylon* (L.)Pers.; G: *Setaria verticillata* (L.) P. Beauv.

**Table 4:** Information on voucher specimens for *Setaria italica* and the other related Poaceae species. GPS coordinates are provided. All samples in this research were collected by H.-S. Lin and deposited in the Institute of Biodiversity, Department of Life Science, National Cheng Kung University, Taiwan.

Pop	Taxon	Collection Sites	GPS Coordinates (N,E)	Collection Number
P1	*Setaria italica* (L.) P. Beauv	Jianshi Township, Hsinchu County, Taiwan	24.675722°,121.208725°	NCKU.S.I.P1001~NCKU.S.I.P10020
P2	*Setaria italica* (L.) P. Beauv	Wufeng township, Hsinchu county, Taiwan	24.567733°,121.142120°	NCKU.S.I.P2001~NCKU.S.I.P20022
P3	*Setaria italica* (L.) P. Beauv	Ren’ai Township, Nantou County, Taiwan	24.012599°, 121.124954°	NCKU.S.I.P3001~NCKU.S.I.P30018
P4	*Setaria italica* (L.) P. Beauv	Yuchi Township, Nantou County, Taiwan	23.891880°, 120.917244°	NCKU.S.I.P4001~NCKU.S.I.P40025
P5	*Setaria italica* (L.) P. Beauv	Xinyi Township, Nantou County, Taiwan	23.621878°, 120.882912°	NCKU.S.I.P5001~NCKU.S.I.P50017
P6	*Setaria italica* (L.) P. Beauv	Yanping Township, Taitung County, Taiwan	2.894283°, 121.062212°	NCKU.S.I.P6001~NCKU.S.I.P60016
P7	*Setaria italica* (L.) P. Beauv	Daren Township, Taitung County, Taiwan	22.269876°, 120.852871°	NCKU.S.I.P7001~NCKU.S.I.P70020
P8	*Setaria italica* (L.) P. Beauv	Shizi Township, Pingtung County, Taiwan	22.350076°, 120.745239°	NCKU.S.I.P8001~NCKU.S.I.P80021
P9	*Setaria italica* (L.) P. Beauv	Laiyi Township, Pingtung County, Taiwan	22.527106°,120.682325°	NCKU.S.I.P9001~NCKU.S.I.P90017
P10	*Setaria italica* (L.) P. Beauv	ManzHou Township, Pingtung County, Taiwan	22.109498°, 120.873299°	NCKU.S.I.P10001~NCKU.S.I.P100016
P11	*Setaria italica* (L.) P. Beauv	Lanyu Township, Taitung County, Taiwan	22.057005°, 121.562519°	NCKU.S.I.P11001~NCKU.S.I.P110023
P12	*Setaria italica* (L.) P. Beauv	Lanyu Township, Taitung County, Taiwan	22.055812°, 121.515269°	NCKU.S.I.P12001~NCKU.S.I.P120018
	*Hygroryza aristata* (Retz.) Nees	Liuying Dist., Tainan City, Taiwan	23.265053°,120.332919°	NCKU.H.A.001~NCKU.H.A.003
	*Setaria plicata* (Lamk.) T cooke	Ren’ai Township, Nantou County, Taiwan	22.272418°,120.843773°	NCKU.S.P.001~NCKU.S.P.003
	*Microstegium Vimineum* (Trin.) A camus	Alishan Township, Chiayi County, Taiwan	23.469417°,120.702517°	NCKU.M.V.001~NCKU.M.V.003
	*Oplimenus compositus* (L.) P. Beauv	Alishan Township, Chiayi County, Taiwan	23.470952°,120.702742°	NCKU.O.C.001~NCKU.O.C.003
	*Cynodon dactylon* (L.) Pers.	East Dist., Tainan City, Taiwan	23.000550°,120.219870°	NCKU.C.D.001~NCKU.C.D.003
	*Setaria verticillata* (L.) P. Beauv.	Annan Dist., Tainan City, Taiwan	23.058302°,120.134146°	NCKU.S.V.001~NCKU.S.V.003
