# Supplementary material for: Development of Simple Sequence Repeats (SSR) Markers in Setaria italica (Poaceae) and Cross-Amplification in Related Species
Source: Int J Mol Sci. 2011 Nov 11;12(11):7835–45. doi: 10.3390/ijms12117835 (PMC3233442; doi:10.3390/ijms12117835)
Supplement: Supplementary file 1 [file ijms-12-07835-s001.pdf]

Table 1S. Results of initial primer screening in twelve populations of *Setaria italica*. Parameters shown for each pair of primer are the number of the samples for each population (N), number of alleles for each population (N_a) and observed heterozygosity (H_o).

	**Hsinchu**						**Nantou**						**Taitung**						**Pingtung**						**Orchid Island**		
	**$P_1(N = 20)$**		**$P_2(N = 22)$**		**$P_3(N = 18)$**		**$P_4(N = 25)$**		**$P_5(N = 17)$**		**$P_6(N = 16)$**		**$P_7(N = 20)$**		**$P_8(N = 21)$**		**$P_9(N = 17)$**		**$P_{10}(N = 16)$**		**$P_{11}(N = 23)$**		**$P_{12}(N = 18)$**	
**Locus**	**N_a**	**H_o**	**N_a**	**H_o**	**N_a**	**H_o**	**N_a**	**H_o**	**N_a**	**H_o**	**N_a**	**H_o**	**N_a**	**H_o**	**N_a**	**H_o**	**N_a**	**H_o**	**N_a**	**H_o**	**N_a**	**H_o**	**N_a**	**H_o**
SITM02	2	0.8	2	0.591	2	0.778	2	0.76	2	0.706	2	0.625	2	0.9	2	0.952	2	0.588	1	0.438	2	0.609	1	0.667
SITM04	1	0.75	1	0.636	1	0.944	1	0.88	2	0.882	2	0.938	2	0.95	2	0.952	2	0.882	1	0.875	1	0.957	1	0.944
SITM05	2	0.9	1	0.864	3	0.944	4	0.84	2	1	2	0.938	1	0.95	1	1	3	0.765	2	1	1	0.652	3	0.778
SITM06	1	0.8	1	0.955	1	0.944	1	0.96	1	0.706	1	0.938	1	0.6	1	0.81	2	0.765	1	0.75	2	0.478	2	0.722
SITM07	1	0.7	3	0.773	1	0.778	2	0.92	2	0.824	1	0.688	2	0.5	2	0.905	1	0.765	3	0.938	3	0.478	1	0.444
SITM09	2	0.45	3	0.909	2	0.5	2	0.84	1	0.529	2	0.875	2	0.9	1	0.857	2	0.882	1	0.813	2	0.913	2	0.611
SITM10	1	0.55	1	0.773	1	0.889	1	0.96	2	0.882	1	0.688	2	0.65	1	1	1	1	3	0.75	3	0.348	1	0.444
SITM11	2	0.35	2	0.545	2	0.722	1	0.96	1	0.647	2	0.688	1	0.9	3	1	1	0.941	2	0.75	1	0.739	1	0.389
SITM14	2	0.85	3	0.591	2	0.833	2	0.88	2	0.529	2	0.813	2	0.75	1	0.952	2	0.882	2	0.688	1	0.739	3	0.889
SITM15	1	0.6	1	0.909	2	1	2	0.64	1	0.882	1	0.813	1	0.65	1	0.857	1	1	2	0.875	1	0.783	2	0.444
SITM17	3	0.75	3	0.545	2	0.889	3	0.96	4	0.824	2	0.625	4	0.55	3	1	1	0.882	2	0.813	2	0.783	4	0.889
SITM18	1	0.25	1	0.773	1	0.611	1	0.88	1	0.882	1	0.688	1	0.7	1	0.857	1	0.941	2	0.688	1	0.739	1	0.444
SITM19	1	0.65	1	0.591	2	0.444	1	0.72	4	0.882	2	0.938	1	0.85	2	1	2	0.941	2	0.75	1	0.913	1	0.722
SITM20	1	1	2	0.818	1	1	1	0.84	1	0.588	1	0.938	1	0.95	1	1	2	0.941	1	0.938	1	0.913	2	0.556
SITM22	3	1	1	0.909	3	0.778	3	0.72	3	0.529	3	0.688	3	0.9	2	0.857	2	0.882	1	0.813	1	0.652	1	0.389
SITM23	2	0.35	2	0.591	3	0.722	2	0.8	2	0.647	2	0.938	2	0.55	2	0.857	2	1	3	0.938	2	0.87	2	0.444
SITM24	1	0.7	1	0.5	2	0.833	2	0.64	1	0.706	1	0.875	1	0.75	1	1	1	0.882	2	0.75	2	0.913	2	0.833
SITM25	2	0.45	2	0.455	2	0.444	2	0.72	2	0.647	1	0.875	2	0.85	2	1	2	1	1	0.938	1	0.652	3	0.389
SITM26	1	0.75	1	0.773	1	0.833	1	0.84	2	0.529	2	0.75	2	0.8	2	0.952	1	0.824	2	0.875	2	0.739	2	0.556
SITM27	2	0.85	1	0.955	1	0.944	2	0.76	1	0.765	1	0.813	1	0.95	2	0.952	2	0.824	1	0.813	1	0.826	1	0.611
SITM28	2	0.3	2	0.5	2	0.889	2	0.92	2	0.529	1	0.875	1	0.8	1	0.905	1	0.824	1	0.875	1	0.913	2	0.667
SITM30	1	0.7	1	1	1	0.611	1	0.72	1	0.824	1	0.938	1	0.75	1	0.857	1	1	1	0.75	1	0.826	1	0.556
SITM32	1	0.9	1	0.591	1	1	1	0.76	3	0.706	1	0.75	1	0.9	1	0.952	2	1	1	0.688	1	0.652	3	0.5
SITM33	2	0.25	2	1	1	0.778	2	0.6	2	0.941	1	0.688	2	0.7	2	0.905	2	0.941	2	0.875	2	0.826	2	0.611

Table 1S. *Cont.*

	Hsinchu						Nantou				Taitung				Pingtung						Orchid Island			
	P_1(N = 20)		P_2(N = 22)		P_3(N = 18)		P_4(N = 25)		P_5(N = 17)		P_6(N = 16)		P_7(N = 20)		P_8(N = 21)		P_9(N = 17)		P_{10}(N = 16)		P_{11}(N = 23)		P_{12}(N = 18)	
**Locus**	N_a	H_o	N_a	H_o	N_a	H_o	N_a	H_o	N_a	H_o	N_a	H_o	N_a	H_o	N_a	H_o	N_a	H_o	N_a	H_o	N_a	H_o	N_a	H_o
SITM34	3	0.7	3	0.545	3	0.778	3	0.92	3	0.824	2	0.813	3	0.85	3	0.952	3	0.824	3	0.688	3	0.696	3	0.778
SITM37	1	0.65	2	0.955	2	1	1	0.76	1	0.882	1	0.938	1	0.6	2	0.952	1	0.941	2	0.938	2	0.87	1	0.833
SITM38	1	0.95	1	0.545	1	1	2	0.92	1	0.588	2	0.688	2	0.95	2	1	2	1	2	0.813	3	0.652	1	0.833
SITM40	1	0.25	1	1	2	0.778	1	0.88	1	0.647	1	0.75	2	0.85	1	0.952	1	0.824	1	0.688	2	0.696	1	0.722
SITM41	1	0.75	1	0.955	2	0.944	1	0.72	2	0.882	1	0.813	2	0.95	1	0.952	1	0.882	1	0.688	1	0.739	1	0.833
SITM42	1	0.9	2	0.591	1	0.5	1	0.76	1	0.824	1	0.625	1	0.55	2	0.952	2	0.941	2	0.875	2	0.87	1	0.889
SITM44	2	1	1	0.955	2	0.5	2	0.76	2	0.588	1	0.875	1	0.8	1	0.857	2	1	1	0.688	1	0.826	2	0.444
SITM46	1	0.65	1	0.591	1	0.5	1	0.76	2	0.824	1	0.688	1	0.75	1	0.857	2	0.882	1	0.688	1	0.652	2	0.833
SITM49	1	0.4	1	0.909	1	1	1	0.68	1	0.706	1	0.875	1	0.9	1	1	2	1	1	0.938	1	0.652	1	0.556
SITM51	1	1	1	0.909	1	0.889	1	0.88	1	0.706	1	0.875	1	0.9	1	1	1	0.882	1	0.875	2	0.696	2	0.889
SITM53	1	0.45	3	0.545	1	0.667	3	0.6	1	0.588	1	0.75	1	0.6	2	0.952	2	0.882	2	0.813	2	0.783	2	0.444
SITM55	2	0.45	2	0.5	1	0.556	1	0.72	1	0.706	1	0.688	2	0.95	2	0.905	2	0.882	2	0.938	2	0.696	2	0.5
SITM57	3	0.55	3	0.455	2	0.667	3	0.64	3	0.765	1	0.75	4	0.55	3	0.952	4	1	2	0.688	2	0.739	1	0.444
SITM59	2	0.6	3	0.545	3	0.944	3	0.84	3	0.588	4	0.563	2	0.95	1	0.952	1	0.882	3	0.688	1	0.826	1	0.5
SITM62	1	0.45	1	0.818	1	0.444	1	0.88	1	0.882	1	0.938	1	0.65	1	0.952	1	0.824	2	0.875	1	0.652	1	0.556
SITM65	1	0.6	1	0.909	1	0.5	1	0.8	1	0.882	1	0.625	1	0.6	2	1	1	0.941	1	0.688	2	0.913	1	0.5
SITM68	2	0.95	2	0.545	2	0.889	2	0.88	2	0.824	2	0.688	2	0.55	3	0.857	2	1	2	0.813	3	0.696	2	0.389
SITM73	2	0.4	1	0.545	2	0.833	2	0.88	1	0.765	2	0.75	2	0.7	3	0.905	3	1	1	0.75	3	0.739	2	0.5
SITM84	1	0.8	3	0.545	1	0.778	2	0.96	1	0.706	1	0.75	2	0.8	1	0.952	2	0.941	2	0.813	1	0.87	1	0.889
SITM86	2	0.25	3	0.682	3	0.556	3	0.6	3	0.882	3	0.563	3	0.9	1	1	1	0.941	3	0.813	2	0.652	1	0.444
SITM91	1	0.45	2	1	1	0.833	1	0.8	2	0.706	1	0.813	1	0.65	1	0.857	1	0.824	1	0.688	1	0.652	2	0.389
